# Bio-inspired Flexible Lateral Line Sensor Based on P(VDF-TrFE)/BTO Nanofiber Mat for Hydrodynamic Perception

**DOI:** 10.3390/s19245384

**Published:** 2019-12-06

**Authors:** Xiaohe Hu, Yonggang Jiang, Zhiqiang Ma, Yuanhang Xu, Deyuan Zhang

**Affiliations:** Institute of Bionic and Micro-Nano Systems, School of Mechanical Engineering and Automation, Beihang University, Beijing 100191, China; huxh0706@buaa.edu.cn (X.H.); mazqbuaa@buaa.edu.cn (Z.M.); xuyuanhang@buaa.edu.cn (Y.X.); zhangdy@buaa.edu.cn (D.Z.)

**Keywords:** lateral line system, biomimetics, nanofiber, flow sensor

## Abstract

Fish and some amphibians can perform a variety of behaviors in confined and harsh environments by employing an extraordinary mechanosensory organ, the lateral line system (LLS). Inspired by the form-function of the LLS, a hydrodynamic artificial velocity sensor (HAVS) was presented in this paper. The sensors featured a polarized poly (vinylidene fluoride-trifluoroethylene) [P(VDF-TrFE)]/barium titanate (BTO) electrospinning nanofiber mat as the sensing layer, a polyimide (PI) film with arrays of circular cavities as the substrate, and a poly(methyl methacrylate) (PMMA) pillar as the cilium. The P(VDF-TrFE)/BTO electrospinning nanofiber mat demonstrated enhanced crystallinity and piezoelectricity compared with the pure P(VDF-TrFE) nanofiber mat. A dipole source was employed to characterize the sensing performance of the fabricated HAVS. The HAVS achieved a velocity detection limit of 0.23 mm/s, superior to the conventional nanofiber mat-based flow sensor. In addition, directivity was feasible for the HAVS, which was in accordance with the simulation results. The proposed bio-inspired flexible lateral line sensor with hydrodynamic perception ability shows promising applications in underwater robotics for real-time flow analysis.

## 1. Introduction

Known for their excellent mechanical sensing capabilities, biological mechanosensitive receptors have promoted the advancement of multifunctional sensors in the fields of biomedical engineering, robotics, and artificial intelligence. Remarkably, crack-shaped slit receptors in the legs of scorpions and spiders, specialized for sensing cyclic vibration in a wide range of frequencies, enable them to extract wake signatures left by the prey [1,2]. Flow-sensitive hair receptors of crickets perceive disturbance in the surroundings via variations in weak airflows [3]. Harbor seals use whiskers to decipher hydrodynamic traces left by surrounding animals [4,5]. Inspired by such biological receptors, much progress has been achieved in the field of artificial sensors [6,7,8].

The lateral line system (LLS) allows fish and some amphibians to perform fundamental behaviors for underwater survival, even in complicated environments [9]. The LLS of fish is composed of non-uniformly distributed hair receptors, called neuromasts, which perceive minute motions in water [10]. According to their functions, neuromasts can be divided into two primary types: superficial neuromasts (SNs) and canal neuromasts (CNs) [11]. SNs are distributed on the surface of the body and are sensitive to flow velocities. In addition, SNs respond differently to stimulation sources from different directions for the deflection of stereocilia inducing depolarization and hydepolarization of the electrical activity in the hair cell, thus providing spatial flow direction information along the skin surface of fish [12,13]. CNs are located in lateral line canals and connect to external flow through a series of pores, assisting fish to identify the pressure distribution in the water [14,15]. 

Inspired by the LLS of fish, various artificial flow velocity sensors have been developed [16]. Flow velocity sensors with different sensing mechanisms, such as piezoresistive, capacitive, optical, hot-wire, and piezoelectric properties, have been proposed [17,18,19,20,21,22,23,24,25,26]. Piezoelectric polymers are flexible and biocompatible compared with inorganic materials, and advantageous as they can be used to develop fully flexible sensors for curved surfaces [27]. Bora et al. developed a flow sensor with an electrospun polyvinylidene fluoride (PVDF) nanofiber mat. However, the flow sensor showed a poor detection limit of 5 mm/s and no directional sensing ability [28]. 

PVDF and its copolymer poly(vinylidene fluoride-trifluoroethylene) [P(VDF-TrFE)] are excellent sensor materials as they provide advantages such as energy conservation, excellent processability, and conformal elasticity [29,30]. The piezoelectricity of the PVDF-based polymers can be improved by incorporating inorganic materials such as ZnO, PZT, barium titanate (BTO), graphene and gold nanoparticles (AuNPs) [31,32,33,34,35]. Moreover, electrospinning is an effective way to obtain highly aligned piezoelectric nanofibers with anisotropic piezoelectricity [36]. In this paper, a hydrodynamic artificial velocity sensor (HAVS) was introduced, mimicking the LLS of fish. A highly aligned P(VDF-TrFE)/BTO piezoelectric nanofiber mat with enhanced crystallinity was used as the sensing layer. The nanofibers mat is sensitive along the alignment direction compared with that perpendicular to the fiber alignment, which provides the HAVS device with directivity. 

## 2. Design of the HAVS

### 2.1. Sensor Structure and Sensing Principle 

**Figure 1:**
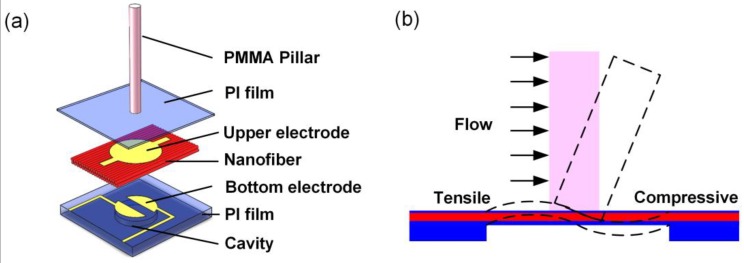
(**a**) A schematic diagram of an individual HAVS; (**b**) Sensing principle of the HAVS device.

The HAVS device inspired by the LLS is composed of a flexible polyimide (PI) substrate with a round cavity, a piezoelectric P(VDF-TrFE)/BTO nanofiber mat, and a high-aspect-ratio poly(methyl methacrylate) (PMMA) pillar, as shown in Figure 1a. A 50-μm-thick commercial PI substrate is fabricated with a round cavity to obtain a sensor diaphragm with a thickness of 20 μm. Two bottom electrodes (Au/Cr) with semicircular shape (diameter of 2 mm) and an interval of 500 μm are situated on the sensor diaphragm. The thickness of the P(VDF-TrFE)/BTO nanofiber mat is 40 μm, and the diameter of the top electrode is 2.4 mm. Another commercial PI film with a thickness of 6 μm is used to encapsulate the device. The diameter of the pillar is optimized by a multiphysics simulation. The simulation results illustrate that the generated signal achieves the maximum when the diameter of the pillar is 600 μm (Figure S1). The commercial available PMMA pillar with a diameter of 500 μm and a height of 5 mm is employed. 

As shown in Figure 1b, when an external flow passes parallel to the sensor surface, a drag force is imparted to the pillar. Due to the rigid connection between the pillar and the PI film, the bending moment is transferred to the bottom membrane inducing the piezoelectric output. 

### 2.2. Directivity of the HAVS 

**Figure 2:**
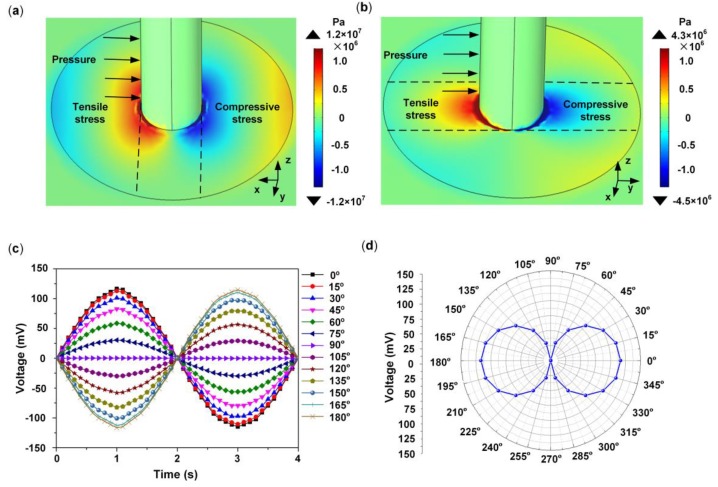
Distribution of stress in the *x* direction when pressure is at 0° (**a**) and at 90° (**b**); (**c**) Relationship between signal outputs and directions of pressure in the time domain; (**d**) Directivity curve of the HAVS device in polar coordinates.

Under an external stimulus, the piezoelectric membrane deforms on one side of the pillar. When the direction of the stimulus changes, stress variation appears on the piezoelectric membrane. Different piezoelectric signals are induced on the piezoelectric membrane, which are obtained from a semicircular electrode. The influence of the flow velocity direction on the piezoelectric output of the HAVS was analyzed by a multiphysics simulation. A sinusoidal uniformly distributed pressure with an amplitude of 200 Pa was exerted on one side of the pillar. The direction of the pressure varied from 0° to 360° (Figure S2). Figure 2a demonstrates the distribution of stress in the *x* direction when the pressure is at 0°. It can be seen that the maximum compressive stress occurs on the right side of the pillar, and the maximum tensile stress occurs on the left. The stress induced piezoelectric signals on both sides of the pillar are obtained by the two semicircular electrodes. Figure 2b demonstrates the stress distribution in the *x* direction when pressure is applied at 90°. The maximum tensile stress and compressive stress appear between the semicircular electrodes, and the induced signal is neutralized on each semicircular electrode. 

Figure 2c illustrates the piezoelectric signals collected from a semicircular electrode by varying the directions of the applied pressures from 0° to 180°. The output signal varies due to the change in the direction of the applied pressure. Maximum peak voltage appears when the pressure is at the 0° and 180° positions. Figure 2d reveals the relationship between the piezoelectric output and the direction of the pressure. The peak amplitudes of the signals can be summarized in a figure of 8 pattern, which demonstrates that the HAVS device is directionally sensitive. 

## 3. Fabrication of the HAVS 

### 3.1. Fabrication Process 

**Figure 3:**
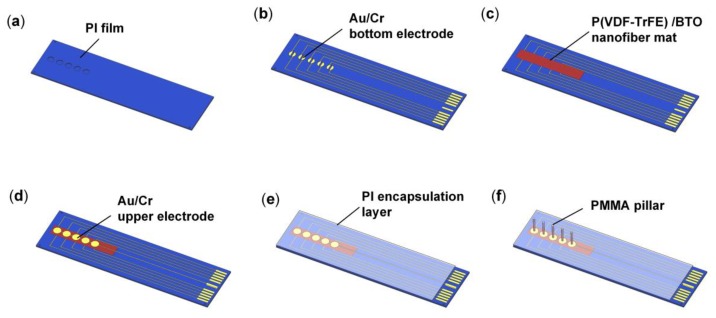
Fabrication process of the bio-inspired lateral line sensor. (**a**) PI film with etched cavities; (**b**) Sputter Au/Cr as the bottom electrodes; (**c**) Transfer the nanofiber mat on the substrate; (**d**) Sputter Au/Cr as the upper electrode; (**e**) Encapsulate the device by a PI film; (**f**) Bond the pillars on the device by epoxy resin to develop the HAVS.

The fabrication process of the HAVS device is illustrated in Figure 3. A commercially available 50-μm-thick PI film was etched to form 30-μm-deep cavities by reactive ion etching with a stencil mask (Figure 3a). After treatment with O_2_ plasma for 3 min, an Au/Cr (160 nm/40 nm) layer was sputtered on the PI film by magnetron sputtering with a stencil mask to form the bottom electrodes of the sensor (Figure 3b). The P(VDF-TrFE)/BTO nanofiber mat was fabricated by a far-field electrospinning method and the detailed electrospinning process was demonstrated in our previous work [33]. The thickness of the nanofiber mat was 40 μm, which was well controlled by adjusting the electrospinning time. The nanofiber mat was annealed in an oven at 135 °C for 2 h. For the poling process, the fiber was sandwiched in PET films with Cu tapes as the electrodes. The device was immersed in silicone oil under an electric field of 8.4 V/μm for 1 h at 80 °C with the same field applied in the cooling run. Schematic diagram of the polarization device is shown in Figure S3.

The fabricated P(VDF-TrFE)/BTO piezoelectric nanofiber mat was then transferred on the PI substrate (Figure 3c). After forming the upper electrodes of Au/Cr (160 nm/40 nm) on the P(VDF-TrFE)/BTO piezoelectric nanofiber mat via the same method as the bottom electrodes (Figure 3d), the laminated structure was encapsulated by a 6-μm-thick PI film (Figure 3e). The fine coaxial cables were connected to the pads by conductive Ag adhesive and then cured on a hot plate at 80 °C for 4 h. A Parylene C layer with a thickness of 7 μm was deposited on the device for waterproofing and electrical isolation. The PMMA pillars were carefully mounted and adhered on the PI film under a digital microscope by epoxy resin. The device was then heat treated for 4 h at 80 °C, guaranteeing that the epoxy resin was completely cured to form a strong contact. Finally, the HAVS device was fabricated as shown in Figure 3f. 

### 3.2. Fabrication Results

**Figure 4:**
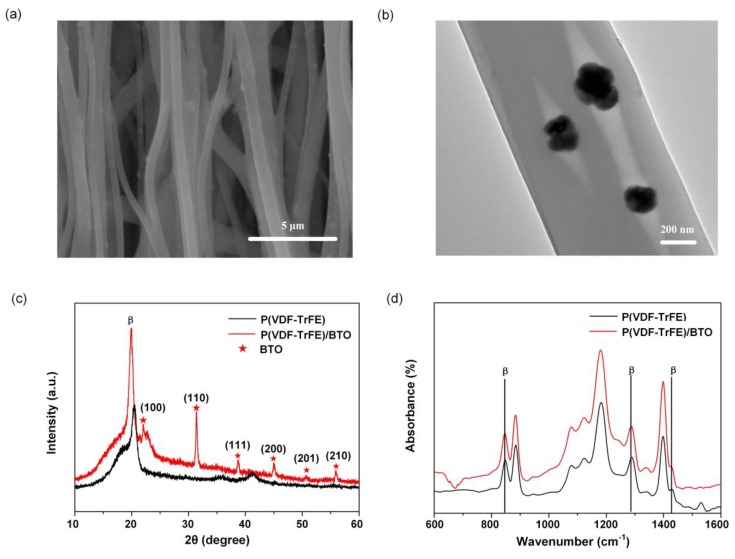
Characterization of the P(VDF-TrFE)/BTO nanofibers. (**a**) SEM image of the P(VDF-TrFE)/BTO nanofiber mat; (**b**) TEM image of a single nanofiber; (**c**) XRD and (**d**) FTIR spectroscopy results for the nanofiber mat.

**Figure 5:**
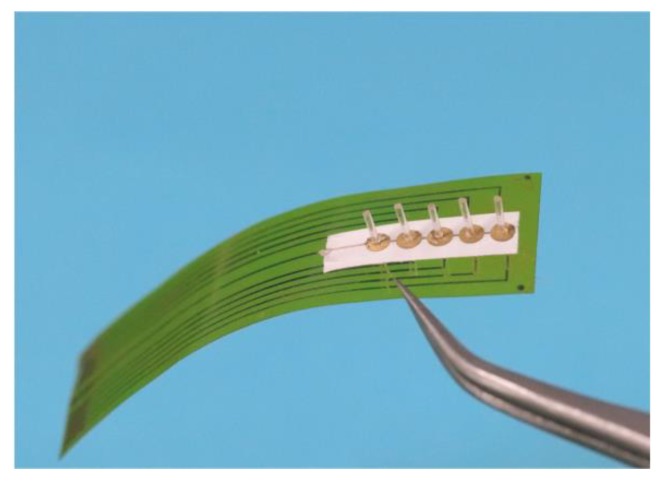
An optical image of the HAVS.

The morphology of the aligned nanofibers is observed by scanning electron microscopy (SEM) and transmission electron microscopy (TEM). Figure 4a shows the SEM image of the nanofibers. The average diameter of the fiber is approximately 500 nm. The fibers present a rough surface, which is attributed to the inclusion of the BTO nanoparticles. The TEM image in Figure 4b illustrates the distribution of the BTO particles (dark grains) in the polymeric matrices. The average diameter of a BTO nanoparticle is approximately 60 nm. X-ray diffraction (XRD) is used to identify the crystalline structures of the nanofiber mat (Figure 4c). The sharpest peak at 19.9° is related to the β phase of the P(VDF-TrFE). The P(VDF-TrFE)/BTO nanofiber mat shows enhanced crystallinity compared to the pristine P(VDF-TrFE) nanofiber mat. The orientation planes of (100), (110), (111), (200), (201) and (210) correspond to the perovskite BTO structure and match well with the standard PDF card JCPDS:31-0174. Fourier transform infrared-transmission (FTIR) spectroscopic analysis is used to identify the functional groups in the nanofiber mat (Figure 4d). The vibration bands at 846 and 1288 cm^−1^ are attributed to the β-crystalline phase. The amount of the β phase is 66.8% for the pristine P(VDF-TrFE) nanofiber mat and 76.3% for the P(VDF-TrFE)/BTO nanofiber mat. The XRD and FTIR results demonstrate that the fibers with BTO show enhanced crystallinity compared with the pristine P(VDF-TrFE) nanofibers. Figure 5 shows the flexible HAVS developed on the PI substrate. 

## 4. Characterization of the HAVS 

### 4.1. Velocity Response of the HAVS 

**Figure 6:**
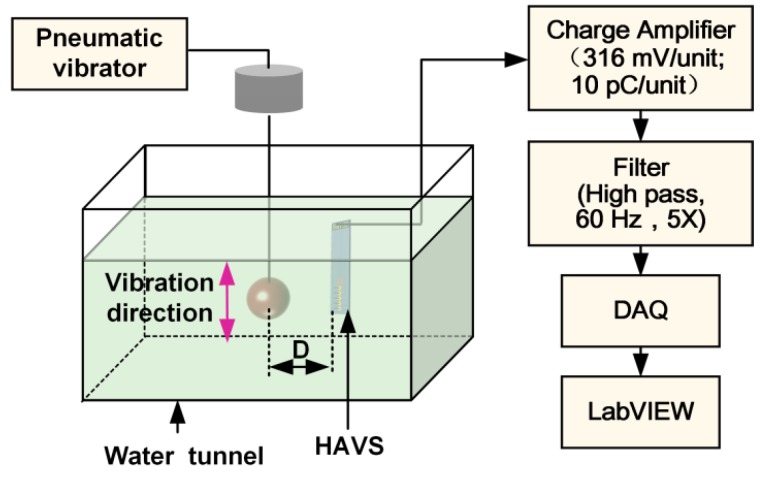
Schematic illustration of the experimental setup for velocity perception.

The velocity response of the HAVS was characterized in a water tunnel of dimensions 0.7 × 0.35 × 0.4 m^3^. The water tunnel was filled with 25-cm-deep water. Schematic diagram of the experimental setup is shown in Figure 6. A hydrodynamic stimulus was generated by a vibrating sphere with a diameter of 15 mm, known as the dipole source, which has been commonly used in studies on artificial LLS [14,31]. The dipole was driven by a pneumatic vibrator through a stainless steel rod, and the vibration amplitude was 1.1 mm. The vibration frequency of the dipole was set at 80 ± 2 Hz in this experiment, and could be adjusted manually. The output signal of the sensor was amplified by a charge amplifier (NEXUS Conditioning Amplifier-2692, Brüel & Kjær, Denmark). The signal was filtered by an adjustable filter (3624, NF Electronic Instruments, USA) with a high pass of 60 Hz and an amplification gain of 5. A data acquisition card (USB-4711, Advantech, China) with a sampling rate of 2000 Hz was used to record the output. The unamplified charge outputs (peak values) of the sensor were employed for statistical analysis. 

The sensor was positioned at the center of the water tunnel immediately beneath the dipole. The dipole vibrated parallel to the direction of the sensor array (perpendicular to the pillar). The flow velocity was adjusted by changing the distance between the dipole center and the apex of the pillar. The velocity detected by the HAVS device was calculated using the following formula [25]:

(1) $$V=\frac{2\pi fsa^{3}}{D},$$

where *f* is the vibration frequency of the dipole, *a* is the diameter of the dipole, *s* is the amplitude of the dipole, and *D* is the distance between the dipole center and the apex of the pillar in the sensor. 

**Figure 7:**
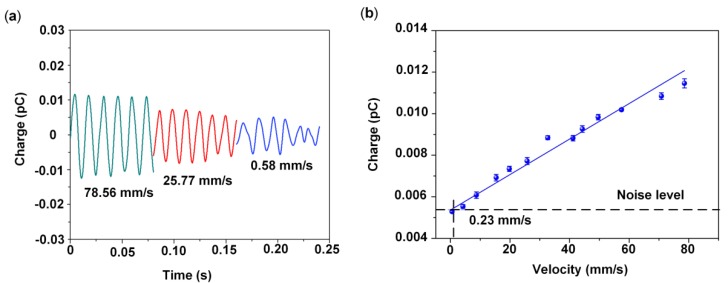
(**a**) Charge output under flow velocities of 78.56, 25.77, and 0.58 mm/s; (**b**) Relationship between charge output and velocity.

Figure 7a shows that the response of the HAVS varied as the velocity was changed to 78.56, 25.77, and 0.58 mm/s. Figure 7b shows the relationship between charge output and velocity. Error bars illustrate the deviation for three repeatable measurements for the same HAVS device. The charge outputs increased with the increase in flow velocities and the sensitivity of the HAVS device was 0.08 pC/(ms^-1^). The detection limit is defined in terms of signal output at the background noise level [37]. The HAVS device demonstrated a velocity detection limit of 0.23 mm/s. Compared with the previous PVDF nanofiber mat-based velocity sensor (detection limit: 5 mm/s) [28], the velocity detection limit in this work is greatly improved, which is probably attributable to the enhanced piezoelectricity of the P(VDF-TrFE)/BTO nanofiber mat after polarization [38].

### 4.2. Directivity Measurement of the HAVS 

**Figure 8:**
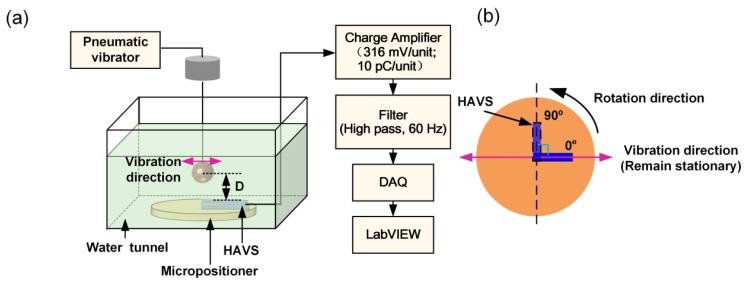
(**a**) A schematic diagram of the experimental setup for directivity detection; (**b**) Testing principle of the HAVS device at 0° and 90°.

The directivity detection of the HAVS device was characterized in the water tunnel, as shown in Figure 8a. The dipole was immersed in water to a depth of 50 mm and vibrated perpendicular to the pillar. The vibration frequency of the dipole was 70 ± 2 Hz. The sensor was positioned on a rotational micropositioner. The distance between the dipole center and the apex of the pillar was fixed as 9.5 mm. The micropositioner stage with the sensor was rotated in 15° increments from 0° to 360°, where 0° represents the direction of fiber alignment along with the direction of dipole vibration. The sensor at the positions of 0° and 90° is illustrated in Figure 8b. The vibration direction of the dipole remained stationary. The velocity at the apex of the pillar, calculated using Equation (1), was found to be 1.9 m/s. The charge outputs of the sensor were averaged over three repeated measurements.

**Figure 9:**
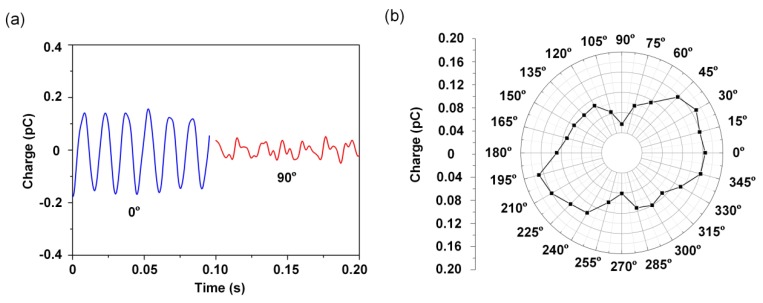
(**a**) Charge output of the HAVS at 0°and 90°; (**b**) Results of the directivity detection.

The sensor output in the time domain is shown in Figure 9a. The charge output of the sensor at 0° was approximately three times higher than that of the sensor at 90°. The figure of 8 pattern of the output signals is displayed in the polar coordinates shown in Figure 9b. The results demonstrated that the HAVS device is directionally sensitive. The different charge outputs can be attributed to the following two factors. Firstly, when the HAVS device was located at 0° and 180°, the fiber orientation was consistent with the direction of dipole vibration. The pillar transmitted the flow field-generated force induced by the dipole vibration to the nanofiber mat. The nanofiber mat is more sensitive to the strain in the fiber alignment direction [36]. However, when the sensor was positioned at 0° and 180°, the fiber orientation may not be exactly the same as the vibration direction of the dipole. As a result, the charge output at 195° was higher than that at 180°. The other factor related to the structure of the bottom electrode, which caused the differences in the charge collection. At 0° and 180°, the stress induced charge output achieved maximum on a semicircular electrode. In the cases of 90° and 270°, the stress induced charge outputs were partially neutralized on a semicircular electrode, which leaded to the decrease in charge output. The fabricated HAVS device opens up possibilities for directional detection. 

## 5. Conclusions

We developed a piezoelectric polymer-based flexible HAVS inspired by the LLS of fish. The sensor featured a polarized P(VDF-TrFE)/BTO electrospinning nanofiber mat and a flexible PI substrate. The XRD and FTIR results demonstrated that the nanofibers with BTO exhibited enhanced crystallinity and piezoelectricity. The HAVS device achieved a lower velocity sensing limitation of 0.23 mm/s, compared to that of the conventional nanofiber mat-based flow sensor (i.e., 5 mm/s). Moreover, the highly aligned P(VDF-TrFE)/BTO nanofiber endowed the flexible HAVS with directivity. Given the excellent sensing performance, the bio-inspired flexible HAVS shows a potential in hydrodynamic images for robotic applications. 

## Supplementary Materials

The following are available online at https://www.mdpi.com/1424-8220/19/24/5384/s1. Figure S1: Relationship between the diameter of the pillar and the generated voltage. Figure S2: Direction of the pressure varied from 0° to 360° in the simulation process. Figure S3: Schematic diagram of the polarization setup.
