# Supplementary material for: Bio-inspired Flexible Lateral Line Sensor Based on P(VDF-TrFE)/BTO Nanofiber Mat for Hydrodynamic Perception
Source: Sensors (Basel). 2019 Dec 6;19(24):5384. doi: 10.3390/s19245384 (PMC6960935; doi:10.3390/s19245384)
Supplement: Supplementary file 1 [file sensors-19-05384-s001.pdf]

Supporting Information

Bio-inspired Flexible Lateral Line Sensor Based on P(VDF-TrFE)/BTO Nanofiber Mat for Hydrodynamic Perception

Xiaohe Hu [1], Yonggang Jiang [1,*], Zhiqiang Ma [1], Yuanhang Xu [1], and Deyuan Zhang [1]

[1] Institute of Bionic and Micro-Nano Systems, School of Mechanical Engineering and Automation, Beihang University, Beijing 100191, China; huxh0706@buaa.edu.cn (X.H.); mazqbuaa@buaa.edu.cn (Z.M.) ; xuyuanhang@buaa.edu.cn (Y.X.); zhangdy@buaa.edu.cn (D.Z.)

* Correspondence: jiangyg@buaa.edu.cn; Tel.: +86-10-8231-6603

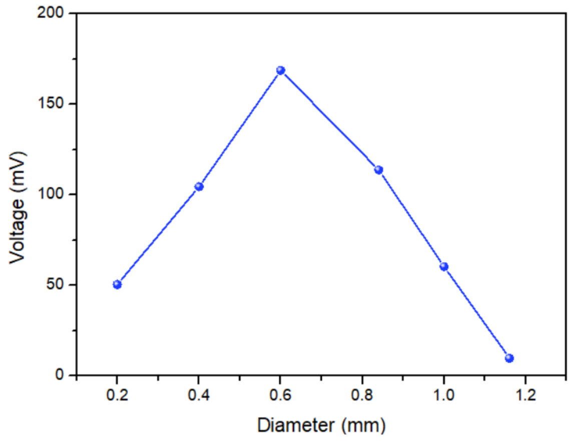

Figure S1. Relationship between the diameter of the pillar and the generated voltage.

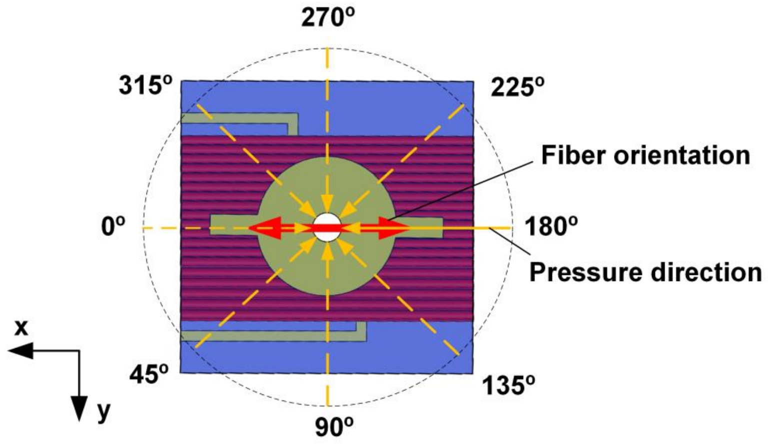

Figure S2. The direction of the pressure varied from 0° to 360° in the simulation process.

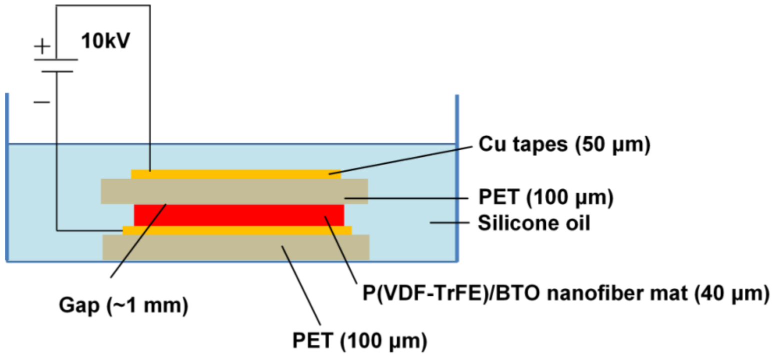

Figure S3. Schematic diagram of the polarization setup.
